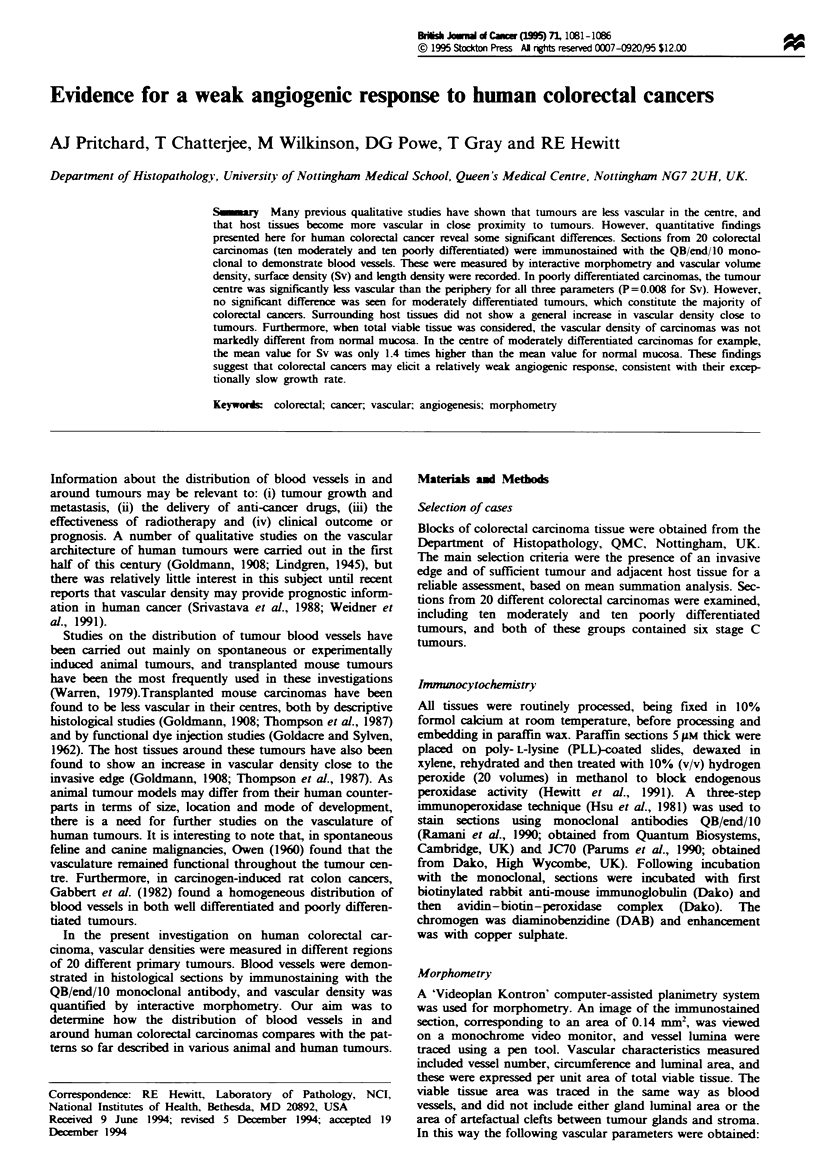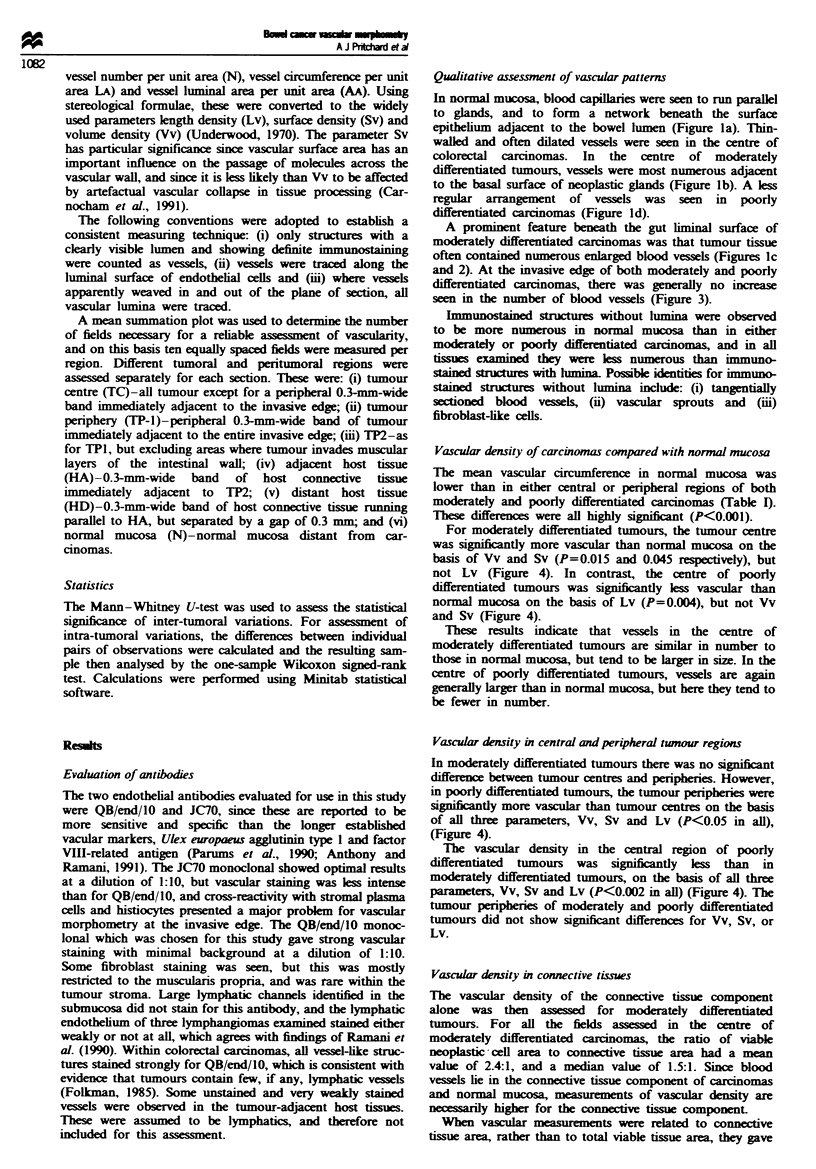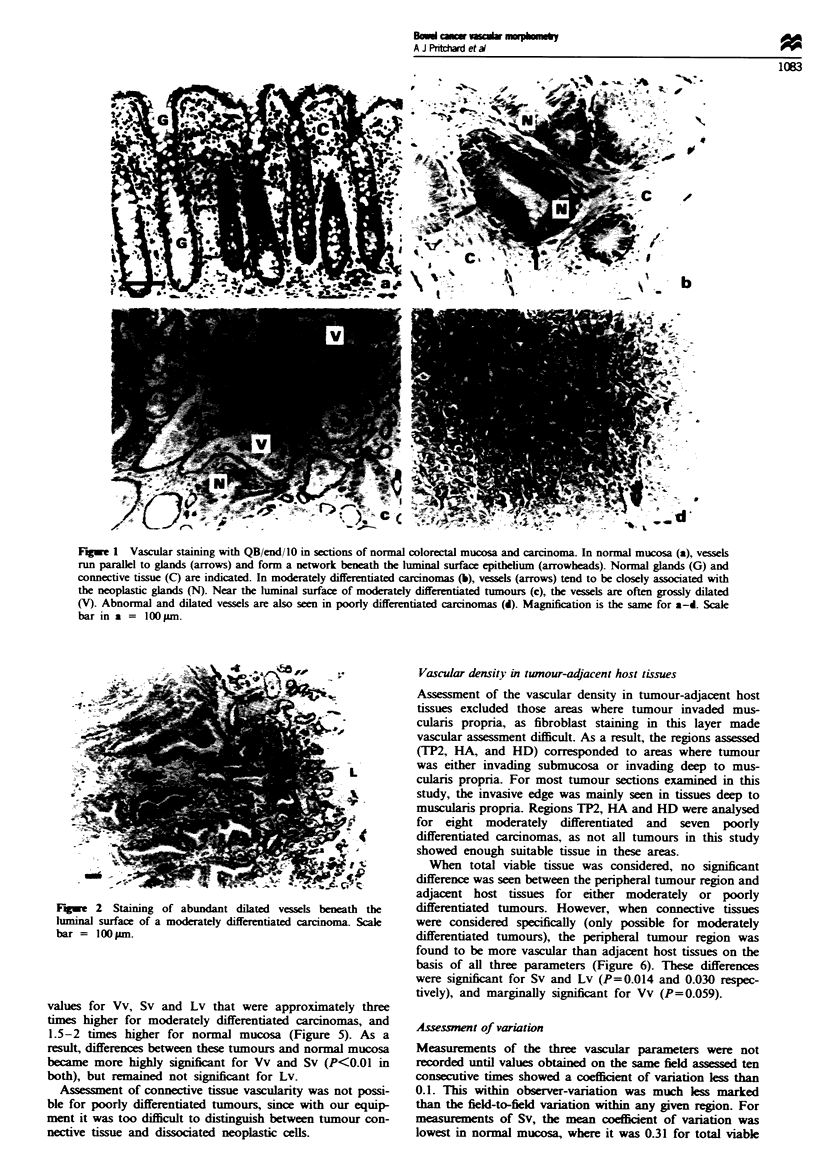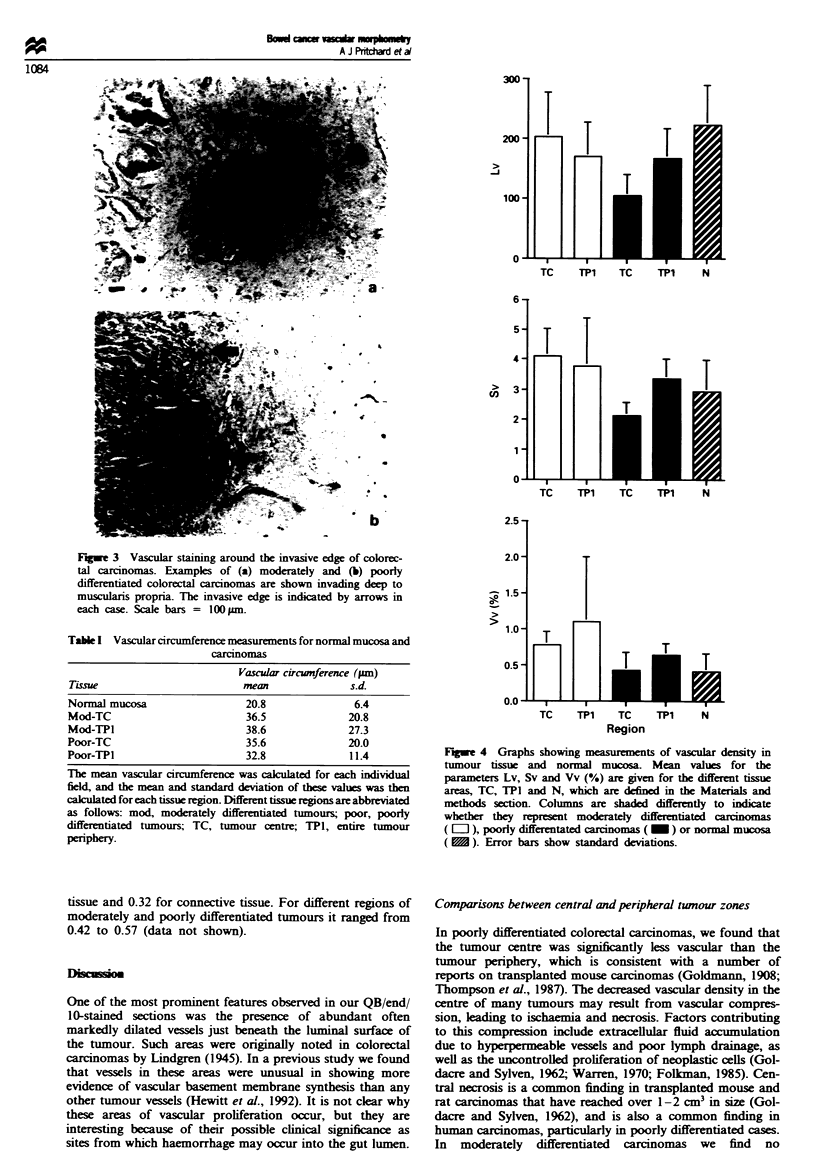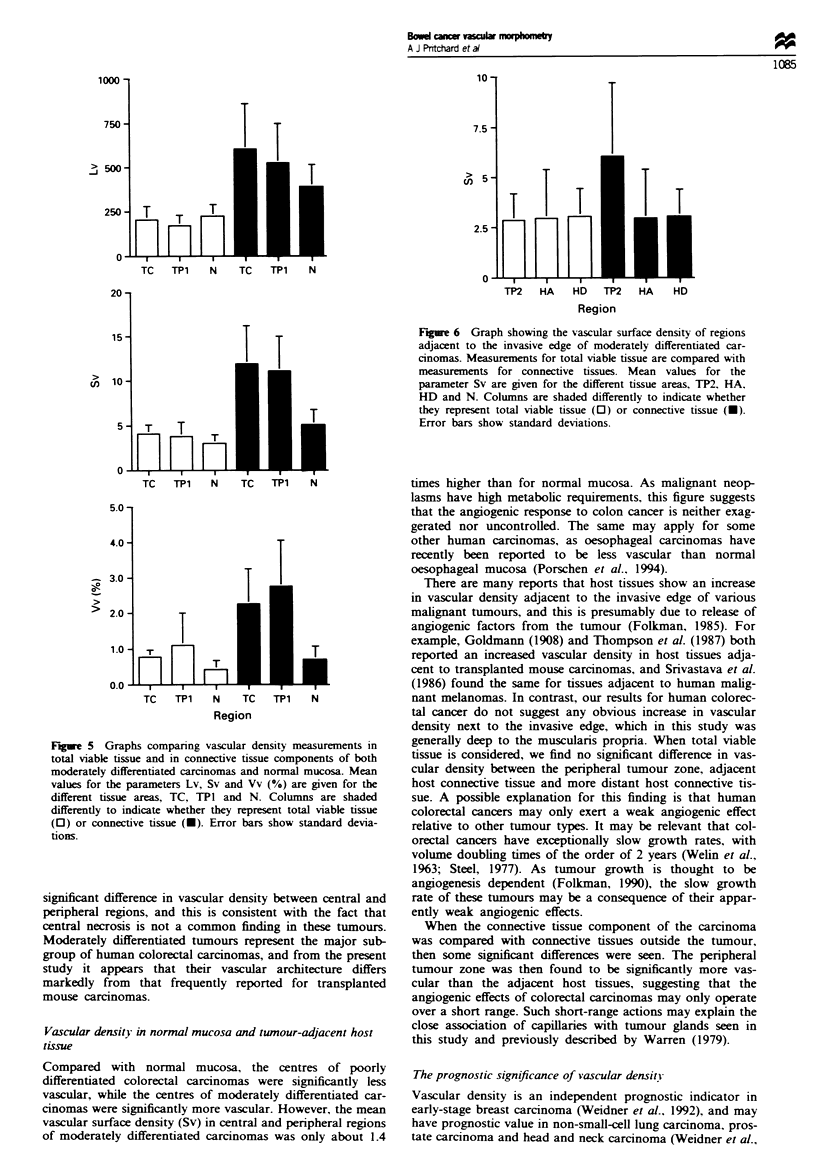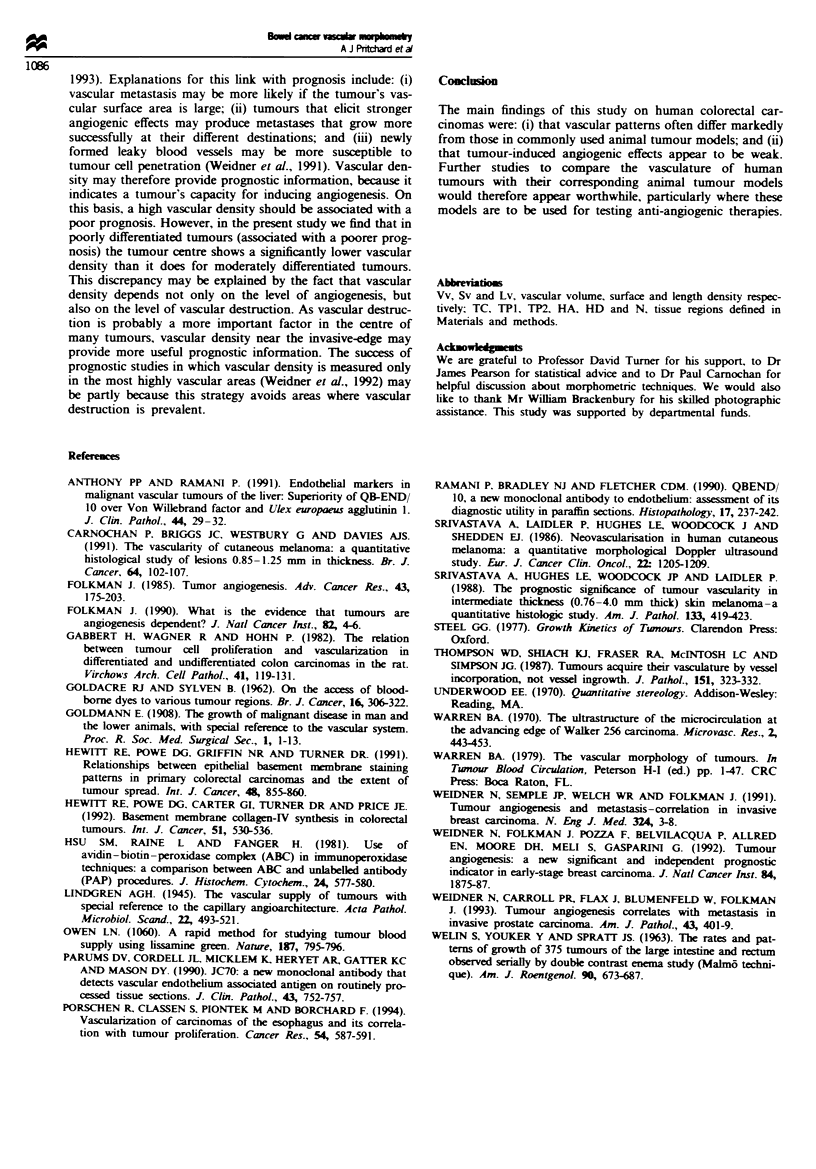# Evidence for a weak angiogenic response to human colorectal cancers.

**DOI:** 10.1038/bjc.1995.209

**Published:** 1995-05

**Authors:** A. J. Pritchard, T. Chatterjee, M. Wilkinson, D. G. Powe, T. Gray, R. E. Hewitt

**Affiliations:** Department of Histopathology, University of Nottingham Medical School, Queen's Medical Centre, UK.

## Abstract

**Images:**


British Journal of Cancer (1995) 71, 1081-1086

© 1995 Stockton Press All rights reserved 0007-0920/95 $12.00

### Evidence for a weak angiogenic response to human colorectal cancers

AJ Pritchard, T Chatterjee, M Wilkinson, DG Powe, T Gray and RE Hewitt

*Department of Histopathology, University of Nottingham Medical School, Queen's Medical Centre, Nottingham NG7 2UH, UK.*

**Summary** Many previous qualitative studies have shown that tumours are less vascular in the centre, and that host tissues become more vascular in close proximity to tumours. However, quantitative findings presented here for human colorectal cancer reveal some significant differences. Sections from 20 colorectal carcinomas (ten moderately and ten poorly differentiated) were immunostained with the QB/end/10 monoclonal to demonstrate blood vessels. These were measured by interactive morphometry and vascular volume density, surface density (Sv) and length density were recorded. In poorly differentiated carcinomas, the tumour centre was significantly less vascular than the periphery for all three parameters (P = 0.008 for Sv). However, no significant difference was seen for moderately differentiated tumours, which constitute the majority of colorectal cancers. Surrounding host tissues did not show a general increase in vascular density close to tumours. Furthermore, when total viable tissue was considered, the vascular density of carcinomas was not markedly different from normal mucosa. In the centre of moderately differentiated carcinomas for example, the mean value for Sv was only 1.4 times higher than the mean value for normal mucosa. These findings suggest that colorectal cancers may elicit a relatively weak angiogenic response, consistent with their exceptionally slow growth rate.

**Keywords:** colorectal; cancer; vascular; angiogenesis; morphometry

Information about the distribution of blood vessels in and around tumours may be relevant to: (i) tumour growth and metastasis, (ii) the delivery of anti-cancer drugs, (iii) the effectiveness of radiotherapy and (iv) clinical outcome or prognosis. A number of qualitative studies on the vascular architecture of human tumours were carried out in the first half of this century (Goldmann, 1908; Lindgren, 1945), but there was relatively little interest in this subject until recent reports that vascular density may provide prognostic information in human cancer (Srivastava *et al.*, 1988; Weidner *et al.*, 1991).

Studies on the distribution of tumour blood vessels have been carried out mainly on spontaneous or experimentally induced animal tumours, and transplanted mouse tumours have been the most frequently used in these investigations (Warren, 1979).Transplanted mouse carcinomas have been found to be less vascular in their centres, both by descriptive histological studies (Goldmann, 1908; Thompson *et al.*, 1987) and by functional dye injection studies (Goldacre and Sylven, 1962). The host tissues around these tumours have also been found to show an increase in vascular density close to the invasive edge (Goldmann, 1908; Thompson *et al.*, 1987). As animal tumour models may differ from their human counterparts in terms of size, location and mode of development, there is a need for further studies on the vasculature of human tumours. It is interesting to note that, in spontaneous feline and canine malignancies, Owen (1960) found that the vasculature remained functional throughout the tumour centre. Furthermore, in carcinogen-induced rat colon cancers, Gabbert *et al.* (1982) found a homogeneous distribution of blood vessels in both well differentiated and poorly differentiated tumours.

In the present investigation on human colorectal carcinoma, vascular densities were measured in different regions of 20 different primary tumours. Blood vessels were demonstrated in histological sections by immunostaining with the QB/end/10 monoclonal antibody, and vascular density was quantified by interactive morphometry. Our aim was to determine how the distribution of blood vessels in and around human colorectal carcinomas compares with the patterns so far described in various animal and human tumours.

Correspondence: RE Hewitt, Laboratory of Pathology, NCI, National Institutes of Health, Bethesda, MD 20892, USA
Received 9 June 1994; revised 5 December 1994; accepted 19 December 1994

### Materials and Methods

### Selection of cases

Blocks of colorectal carcinoma tissue were obtained from the Department of Histopathology, QMC, Nottingham, UK. The main selection criteria were the presence of an invasive edge and of sufficient tumour and adjacent host tissue for a reliable assessment, based on mean summation analysis. Sections from 20 different colorectal carcinomas were examined, including ten moderately and ten poorly differentiated tumours, and both of these groups contained six stage C tumours.

### Immunocytochemistry

All tissues were routinely processed, being fixed in 10% formol calcium at room temperature, before processing and embedding in paraffin wax. Paraffin sections 5 μM thick were placed on poly-L-lysine (PLL)-coated slides, dewaxed in xylene, rehydrated and then treated with 10% (v/v) hydrogen peroxide (20 volumes) in methanol to block endogenous peroxidase activity (Hewitt *et al.*, 1991). A three-step immunoperoxidase technique (Hsu *et al.*, 1981) was used to stain sections using monoclonal antibodies QB/end/10 (Ramani *et al.*, 1990; obtained from Quantum Biosystems, Cambridge, UK) and JC70 (Parums *et al.*, 1990; obtained from Dako, High Wycombe, UK). Following incubation with the monoclonal, sections were incubated with first biotinylated rabbit anti-mouse immunoglobulin (Dako) and then avidin–biotin–peroxidase complex (Dako). The chromogen was diaminobenzidine (DAB) and enhancement was with copper sulphate.

### Morphometry

A 'Videoplan Kontron' computer-assisted planimetry system was used for morphometry. An image of the immunostained section, corresponding to an area of 0.14 $mm^2$, was viewed on a monochrome video monitor, and vessel lumina were traced using a pen tool. Vascular characteristics measured included vessel number, circumference and luminal area, and these were expressed per unit area of total viable tissue. The viable tissue area was traced in the same way as blood vessels, and did not include either gland luminal area or the area of artefactual clefts between tumour glands and stroma. In this way the following vascular parameters were obtained:

vessel number per unit area (N), vessel circumference per unit area LA) and vessel luminal area per unit area (AA). Using stereological formulae, these were converted to the widely used parameters length density (Lv), surface density (Sv) and volume density (Vv) (Underwood, 1970). The parameter Sv has particular significance since vascular surface area has an important influence on the passage of molecules across the vascular wall, and since it is less likely than Vv to be affected by artefactual vascular collapse in tissue processing (Carnocham *et al*., 1991).

The following conventions were adopted to establish a consistent measuring technique: (i) only structures with a clearly visible lumen and showing definite immunostaining were counted as vessels, (ii) vessels were traced along the luminal surface of endothelial cells and (iii) where vessels apparently weaved in and out of the plane of section, all vascular lumina were traced.

A mean summation plot was used to determine the number of fields necessary for a reliable assessment of vascularity, and on this basis ten equally spaced fields were measured per region. Different tumoral and peritumoral regions were assessed separately for each section. These were: (i) tumour centre (TC)-all tumour except for a peripheral 0.3-mm-wide band immediately adjacent to the invasive edge; (ii) tumour periphery (TP-1)-peripheral 0.3-mm-wide band of tumour immediately adjacent to the entire invasive edge; (iii) TP2-as for TP1, but excluding areas where tumour invades muscular layers of the intestinal wall; (iv) adjacent host tissue (HA)-0.3-mm-wide band of host connective tissue immediately adjacent to TP2; (v) distant host tissue (HD)-0.3-mm-wide band of host connective tissue running parallel to HA, but separated by a gap of 0.3 mm; and (vi) normal mucosa (N)-normal mucosa distant from carcinomas.

### *Statistics*

The Mann-Whitney *U*-test was used to assess the statistical significance of inter-tumoral variations. For assessment of intra-tumoral variations, the differences between individual pairs of observations were calculated and the resulting sample then analysed by the one-sample Wilcoxon signed-rank test. Calculations were performed using Minitab statistical software.

### Results

### *Evaluation of antibodies*

The two endothelial antibodies evaluated for use in this study were QB/end/10 and JC70, since these are reported to be more sensitive and specific than the longer established vacular markers, *Ulex europaeus* agglutinin type 1 and factor VIII-related antigen (Parums *et al*., 1990; Anthony and Ramani, 1991). The JC70 monoclonal showed optimal results at a dilution of 1:10, but vascular staining was less intense than for QB/end/10, and cross-reactivity with stromal plasma cells and histiocytes presented a major problem for vascular morphometry at the invasive edge. The QB/end/10 monoclonal which was chosen for this study gave strong vascular staining with minimal background at a dilution of 1:10. Some fibroblast staining was seen, but this was mostly restricted to the muscularis propria, and was rare within the tumour stroma. Large lymphatic channels identified in the submucosa did not stain for this antibody, and the lymphatic endothelium of three lymphangiomas examined stained either weakly or not at all, which agrees with findings of Ramani *et al*. (1990). Within colorectal carcinomas, all vessel-like structures stained strongly for QB/end/10, which is consistent with evidence that tumours contain few, if any, lymphatic vessels (Folkman, 1985). Some unstained and very weakly stained vessels were observed in the tumour-adjacent host tissues. These were assumed to be lymphatics, and therefore not included for this assessment.

### *Qualitative assessment of vascular patterns*

In normal mucosa, blood capillaries were seen to run parallel to glands, and to form a network beneath the surface epithelium adjacent to the bowel lumen (Figure 1a). Thin-walled and often dilated vessels were seen in the centre of colorectal carcinomas. In the centre of moderately differentiated tumours, vessels were most numerous adjacent to the basal surface of neoplastic glands (Figure 1b). A less regular arrangement of vessels was seen in poorly differentiated carcinomas (Figure 1d).

A prominent feature beneath the gut liminal surface of moderately differentiated carcinomas was that tumour tissue often contained numerous enlarged blood vessels (Figures 1c and 2). At the invasive edge of both moderately and poorly differentiated carcinomas, there was generally no increase seen in the number of blood vessels (Figure 3).

Immunostained structures without lumina were observed to be more numerous in normal mucosa than in either moderately or poorly differentiated carcinomas, and in all tissues examined they were less numerous than immunostained structures with lumina. Possible identities for immunostained structures without lumina include: (i) tangentially sectioned blood vessels, (ii) vascular sprouts and (iii) fibroblast-like cells.

### *Vascular density of carcinomas compared with normal mucosa*

The mean vascular circumference in normal mucosa was lower than in either central or peripheral regions of both moderately and poorly differentiated carcinomas (Table I). These differences were all highly significant ($P<0.001$).

For moderately differentiated tumours, the tumour centre was significantly more vascular than normal mucosa on the basis of Vv and Sv ($P=0.015$ and 0.045 respectively), but not Lv (Figure 4). In contrast, the centre of poorly differentiated tumours was significantly less vascular than normal mucosa on the basis of Lv ($P=0.004$), but not Vv and Sv (Figure 4).

These results indicate that vessels in the centre of moderately differentiated tumours are similar in number to those in normal mucosa, but tend to be larger in size. In the centre of poorly differentiated tumours, vessels are again generally larger than in normal mucosa, but here they tend to be fewer in number.

### *Vascular density in central and peripheral tumour regions*

In moderately differentiated tumours there was no significant difference between tumour centres and peripheries. However, in poorly differentiated tumours, the tumour peripheries were significantly more vascular than tumour centres on the basis of all three parameters, Vv, Sv and Lv ($P<0.05$ in all), (Figure 4).

The vascular density in the central region of poorly differentiated tumours was significantly less than in moderately differentiated tumours, on the basis of all three parameters, Vv, Sv and Lv ($P<0.002$ in all) (Figure 4). The tumour peripheries of moderately and poorly differentiated tumours did not show significant differences for Vv, Sv, or Lv.

### *Vascular density in connective tissues*

The vascular density of the connective tissue component alone was then assessed for moderately differentiated tumours. For all the fields assessed in the centre of moderately differentiated carcinomas, the ratio of viable neoplastic cell area to connective tissue area had a mean value of 2.4:1, and a median value of 1.5:1. Since blood vessels lie in the connective tissue component of carcinomas and normal mucosa, measurements of vascular density are necessarily higher for the connective tissue component.

When vascular measurements were related to connective tissue area, rather than to total viable tissue area, they gave

**Figure 1** Vascular staining with QB/end/10 in sections of normal colorectal mucosa and carcinoma. In normal mucosa (**a**), vessels run parallel to glands (arrows) and form a network beneath the luminal surface epithelium (arrowheads). Normal glands (G) and connective tissue (C) are indicated. In moderately differentiated carcinomas (**b**), vessels (arrows) tend to be closely associated with the neoplastic glands (N). Near the luminal surface of moderately differentiated tumours (**c**), the vessels are often grossly dilated (V). Abnormal and dilated vessels are also seen in poorly differentiated carcinomas (**d**). Magnification is the same for **a**–**d**. Scale bar in **a** = 100 μm.

**Figure 2** Staining of abundant dilated vessels beneath the luminal surface of a moderately differentiated carcinoma. Scale bar = 100 μm.

values for Vv, Sv and Lv that were approximately three times higher for moderately differentiated carcinomas, and 1.5–2 times higher for normal mucosa (Figure 5). As a result, differences between these tumours and normal mucosa became more highly significant for Vv and Sv ($P<0.01$ in both), but remained not significant for Lv.

Assessment of connective tissue vascularity was not possible for poorly differentiated tumours, since with our equipment it was too difficult to distinguish between tumour connective tissue and dissociated neoplastic cells.

### *Vascular density in tumour-adjacent host tissues*

Assessment of the vascular density in tumour-adjacent host tissues excluded those areas where tumour invaded muscularis propria, as fibroblast staining in this layer made vascular assessment difficult. As a result, the regions assessed (TP2, HA, and HD) corresponded to areas where tumour was either invading submucosa or invading deep to muscularis propria. For most tumour sections examined in this study, the invasive edge was mainly seen in tissues deep to muscularis propria. Regions TP2, HA and HD were analysed for eight moderately differentiated and seven poorly differentiated carcinomas, as not all tumours in this study showed enough suitable tissue in these areas.

When total viable tissue was considered, no significant difference was seen between the peripheral tumour region and adjacent host tissues for either moderately or poorly differentiated tumours. However, when connective tissues were considered specifically (only possible for moderately differentiated tumours), the peripheral tumour region was found to be more vascular than adjacent host tissues on the basis of all three parameters (Figure 6). These differences were significant for Sv and Lv ($P=0.014$ and 0.030 respectively), and marginally significant for Vv ($P=0.059$).

### *Assessment of variation*

Measurements of the three vascular parameters were not recorded until values obtained on the same field assessed ten consecutive times showed a coefficient of variation less than 0.1. This within observer-variation was much less marked than the field-to-field variation within any given region. For measurements of Sv, the mean coefficient of variation was lowest in normal mucosa, where it was 0.31 for total viable

**Figure 3** Vascular staining around the invasive edge of colorectal carcinomas. Examples of (**a**) moderately and (**b**) poorly differentiated colorectal carcinomas are shown invading deep to muscularis propria. The invasive edge is indicated by arrows in each case. Scale bars = 100 µm.

**Table I** Vascular circumference measurements for normal mucosa and carcinomas

| *Tissue* | *Vascular circumference (µm)* *mean* | *s.d.* |
|---|---|---|
| Normal mucosa | 20.8 | 6.4 |
| Mod-TC | 36.5 | 20.8 |
| Mod-TP1 | 38.6 | 27.3 |
| Poor-TC | 35.6 | 20.0 |
| Poor-TP1 | 32.8 | 11.4 |

The mean vascular circumference was calculated for each individual field, and the mean and standard deviation of these values was then calculated for each tissue region. Different tissue regions are abbreviated as follows: mod, moderately differentiated tumours; poor, poorly differentiated tumours; TC, tumour centre; TP1, entire tumour periphery.

**Figure 4** Graphs showing measurements of vascular density in tumour tissue and normal mucosa. Mean values for the parameters Lv, Sv and Vv (%) are given for the different tissue areas, TC, TP1 and N, which are defined in the Materials and methods section. Columns are shaded differently to indicate whether they represent moderately differentiated carcinomas ( □ ), poorly differentated carcinomas ( ■ ) or normal mucosa ( ▨ ). Error bars show standard deviations.

tissue and 0.32 for connective tissue. For different regions of moderately and poorly differentiated tumours it ranged from 0.42 to 0.57 (data not shown).

### Discussion

One of the most prominent features observed in our QB/end/10-stained sections was the presence of abundant often markedly dilated vessels just beneath the luminal surface of the tumour. Such areas were originally noted in colorectal carcinomas by Lindgren (1945). In a previous study we found that vessels in these areas were unusual in showing more evidence of vascular basement membrane synthesis than any other tumour vessels (Hewitt *et al.*, 1992). It is not clear why these areas of vascular proliferation occur, but they are interesting because of their possible clinical significance as sites from which haemorrhage may occur into the gut lumen.

*Comparisons between central and peripheral tumour zones*

In poorly differentiated colorectal carcinomas, we found that the tumour centre was significantly less vascular than the tumour periphery, which is consistent with a number of reports on transplanted mouse carcinomas (Goldmann, 1908; Thompson *et al.*, 1987). The decreased vascular density in the centre of many tumours may result from vascular compression, leading to ischaemia and necrosis. Factors contributing to this compression include extracellular fluid accumulation due to hyperpermeable vessels and poor lymph drainage, as well as the uncontrolled proliferation of neoplastic cells (Goldacre and Sylven, 1962; Warren, 1970; Folkman, 1985). Central necrosis is a common finding in transplanted mouse and rat carcinomas that have reached over 1–2 $cm^3$ in size (Goldacre and Sylven, 1962), and is also a common finding in human carcinomas, particularly in poorly differentiated cases. In moderately differentiated carcinomas we find no

**Figure 5** Graphs comparing vascular density measurements in total viable tissue and in connective tissue components of both moderately differentiated carcinomas and normal mucosa. Mean values for the parameters Lv, Sv and Vv (%) are given for the different tissue areas, TC, TP1 and N. Columns are shaded differently to indicate whether they represent total viable tissue (□) or connective tissue (■). Error bars show standard deviations.

**Figure 6** Graph showing the vascular surface density of regions adjacent to the invasive edge of moderately differentiated carcinomas. Measurements for total viable tissue are compared with measurements for connective tissues. Mean values for the parameter Sv are given for the different tissue areas, TP2, HA, HD and N. Columns are shaded differently to indicate whether they represent total viable tissue (□) or connective tissue (■). Error bars show standard deviations.

significant difference in vascular density between central and peripheral regions, and this is consistent with the fact that central necrosis is not a common finding in these tumours. Moderately differentiated tumours represent the major subgroup of human colorectal carcinomas, and from the present study it appears that their vascular architecture differs markedly from that frequently reported for transplanted mouse carcinomas.

*Vascular density in normal mucosa and tumour-adjacent host tissue*

Compared with normal mucosa, the centres of poorly differentiated colorectal carcinomas were significantly less vascular, while the centres of moderately differentiated carcinomas were significantly more vascular. However, the mean vascular surface density (Sv) in central and peripheral regions of moderately differentiated carcinomas was only about 1.4 times higher than for normal mucosa. As malignant neoplasms have high metabolic requirements, this figure suggests that the angiogenic response to colon cancer is neither exaggerated nor uncontrolled. The same may apply for some other human carcinomas, as oesophageal carcinomas have recently been reported to be less vascular than normal oesophageal mucosa (Porschen *et al.*, 1994).

There are many reports that host tissues show an increase in vascular density adjacent to the invasive edge of various malignant tumours, and this is presumably due to release of angiogenic factors from the tumour (Folkman, 1985). For example, Goldmann (1908) and Thompson *et al.* (1987) both reported an increased vascular density in host tissues adjacent to transplanted mouse carcinomas, and Srivastava *et al.* (1986) found the same for tissues adjacent to human malignant melanomas. In contrast, our results for human colorectal cancer do not suggest any obvious increase in vascular density next to the invasive edge, which in this study was generally deep to the muscularis propria. When total viable tissue is considered, we find no significant difference in vascular density between the peripheral tumour zone, adjacent host connective tissue and more distant host connective tissue. A possible explanation for this finding is that human colorectal cancers may only exert a weak angiogenic effect relative to other tumour types. It may be relevant that colorectal cancers have exceptionally slow growth rates, with volume doubling times of the order of 2 years (Welin *et al.*, 1963; Steel, 1977). As tumour growth is thought to be angiogenesis dependent (Folkman, 1990), the slow growth rate of these tumours may be a consequence of their apparently weak angiogenic effects.

When the connective tissue component of the carcinoma was compared with connective tissues outside the tumour, then some significant differences were seen. The peripheral tumour zone was then found to be significantly more vascular than the adjacent host tissues, suggesting that the angiogenic effects of colorectal carcinomas may only operate over a short range. Such short-range actions may explain the close association of capillaries with tumour glands seen in this study and previously described by Warren (1979).

*The prognostic significance of vascular density*

Vascular density is an independent prognostic indicator in early-stage breast carcinoma (Weidner *et al.*, 1992), and may have prognostic value in non-small-cell lung carcinoma, prostate carcinoma and head and neck carcinoma (Weidner *et al.*,

1993). Explanations for this link with prognosis include: (i) vascular metastasis may be more likely if the tumour's vascular surface area is large; (ii) tumours that elicit stronger angiogenic effects may produce metastases that grow more successfully at their different destinations; and (iii) newly formed leaky blood vessels may be more susceptible to tumour cell penetration (Weidner *et al.*, 1991). Vascular density may therefore provide prognostic information, because it indicates a tumour's capacity for inducing angiogenesis. On this basis, a high vascular density should be associated with a poor prognosis. However, in the present study we find that in poorly differentiated tumours (associated with a poorer prognosis) the tumour centre shows a significantly lower vascular density than it does for moderately differentiated tumours. This discrepancy may be explained by the fact that vascular density depends not only on the level of angiogenesis, but also on the level of vascular destruction. As vascular destruction is probably a more important factor in the centre of many tumours, vascular density near the invasive-edge may provide more useful prognostic information. The success of prognostic studies in which vascular density is measured only in the most highly vascular areas (Weidner *et al.*, 1992) may be partly because this strategy avoids areas where vascular destruction is prevalent.

### Conclusion

The main findings of this study on human colorectal carcinomas were: (i) that vascular patterns often differ markedly from those in commonly used animal tumour models; and (ii) that tumour-induced angiogenic effects appear to be weak. Further studies to compare the vasculature of human tumours with their corresponding animal tumour models would therefore appear worthwhile, particularly where these models are to be used for testing anti-angiogenic therapies.

### Abbreviations

Vv, Sv and Lv, vascular volume, surface and length density respectively; TC, TP1, TP2, HA, HD and N, tissue regions defined in Materials and methods.

### Acknowledgements

We are grateful to Professor David Turner for his support, to Dr James Pearson for statistical advice and to Dr Paul Carnochan for helpful discussion about morphometric techniques. We would also like to thank Mr William Brackenbury for his skilled photographic assistance. This study was supported by departmental funds.

### References

ANTHONY PP AND RAMANI P. (1991). Endothelial markers in malignant vascular tumours of the liver: Superiority of QB-END/10 over Von Willebrand factor and *Ulex europaeus* agglutinin 1. *J. Clin. Pathol.*, **44**, 29-32.

CARNOCHAN P. BRIGGS JC, WESTBURY G AND DAVIES AJS. (1991). The vascularity of cutaneous melanoma: a quantitative histological study of lesions 0.85-1.25 mm in thickness. *Br. J. Cancer*, **64**, 102-107.

FOLKMAN J. (1985). Tumor angiogenesis. *Adv. Cancer Res.*, **43**, 175-203.

FOLKMAN J. (1990). What is the evidence that tumours are angiogenesis dependent? *J. Natl Cancer Inst.*, **82**, 4-6.

GABBERT H. WAGNER R AND HOHN P. (1982). The relation between tumour cell proliferation and vascularization in differentiated and undifferentiated colon carcinomas in the rat. *Virchows Arch. Cell Pathol.*, **41**, 119-131.

GOLDACRE RJ AND SYLVEN B. (1962). On the access of blood-borne dyes to various tumour regions. *Br. J. Cancer*, **16**, 306-322.

GOLDMANN E. (1908). The growth of malignant disease in man and the lower animals, with special reference to the vascular system. *Proc. R. Soc. Med. Surgical Sec.*, **1**, 1-13.

HEWITT RE, POWE DG, GRIFFIN NR AND TURNER DR. (1991). Relationships between epithelial basement membrane staining patterns in primary colorectal carcinomas and the extent of tumour spread. *Int. J. Cancer*, **48**, 855-860.

HEWITT RE, POWE DG, CARTER GI, TURNER DR AND PRICE JE. (1992). Basement membrane collagen-IV synthesis in colorectal tumours. *Int. J. Cancer*, **51**, 530-536.

HSU SM, RAINE L AND FANGER H. (1981). Use of avidin-biotin-peroxidase complex (ABC) in immunoperoxidase techniques: a comparison between ABC and unlabelled antibody (PAP) procedures. *J. Histochem. Cytochem.*, **24**, 577-580.

LINDGREN AGH. (1945). The vascular supply of tumours with special reference to the capillary angioarchitecture. *Acta Pathol. Microbiol. Scand.*, **22**, 493-521.

OWEN LN. (1060). A rapid method for studying tumour blood supply using lissamine green. *Nature*, **187**, 795-796.

PARUMS DV, CORDELL JL, MICKLEM K, HERYET AR, GATTER KC AND MASON DY. (1990). JC70: a new monoclonal antibody that detects vascular endothelium associated antigen on routinely processed tissue sections. *J. Clin. Pathol.*, **43**, 752-757.

PORSCHEN R, CLASSEN S, PIONTEK M AND BORCHARD F. (1994). Vascularization of carcinomas of the esophagus and its correlation with tumour proliferation. *Cancer Res.*, **54**, 587-591.

RAMANI P, BRADLEY NJ AND FLETCHER CDM. (1990). QBEND/10, a new monoclonal antibody to endothelium: assessment of its diagnostic utility in paraffin sections. *Histopathology*, **17**, 237-242.

SRIVASTAVA A, LAIDLER P, HUGHES LE, WOODCOCK J AND SHEDDEN EJ. (1986). Neovascularisation in human cutaneous melanoma: a quantitative morphological Doppler ultrasound study. *Eur. J. Cancer Clin. Oncol.*, **22**: 1205-1209.

SRIVASTAVA A, HUGHES LE, WOODCOCK JP AND LAIDLER P. (1988). The prognostic significance of tumour vascularity in intermediate thickness (0.76-4.0 mm thick) skin melanoma-a quantitative histologic study. *Am. J. Pathol.* **133**, 419-423.

STEEL GG. (1977). *Growth Kinetics of Tumours*. Clarendon Press: Oxford.

THOMPSON WD, SHIACH KJ, FRASER RA, McINTOSH LC AND SIMPSON JG. (1987). Tumours acquire their vasculature by vessel incorporation, not vessel ingrowth. *J. Pathol.*, **151**, 323-332.

UNDERWOOD EE. (1970). *Quantitative stereology*. Addison-Wesley: Reading, MA.

WARREN BA. (1970). The ultrastructure of the microcirculation at the advancing edge of Walker 256 carcinoma. *Microvasc. Res.*, **2**, 443-453.

WARREN BA. (1979). The vascular morphology of tumours. *In Tumour Blood Circulation*, Peterson H-I (ed.) pp. 1-47. CRC Press: Boca Raton, FL.

WEIDNER N, SEMPLE JP, WELCH WR AND FOLKMAN J. (1991). Tumour angiogenesis and metastasis-correlation in invasive breast carcinoma. *N. Eng J. Med.* **324**, 3-8.

WEIDNER N, FOLKMAN J. POZZA F, BELVILACQUA P, ALLRED EN, MOORE DH, MELI S, GASPARINI G. (1992). Tumour angiogenesis: a new significant and independent prognostic indicator in early-stage breast carcinoma. *J. Natl Cancer Inst.* **84**, 1875-87.

WEIDNER N, CARROLL PR, FLAX J, BLUMENFELD W, FOLKMAN J. (1993). Tumour angiogenesis correlates with metastasis in invasive prostate carcinoma. *Am. J. Pathol.*, **43**, 401-9.

WELIN S, YOUKER Y AND SPRATT JS. (1963). The rates and patterns of growth of 375 tumours of the large intestine and rectum observed serially by double contrast enema study (Malmö technique). *Am. J. Roentgenol.* **90**, 673-687.